# Transient Arrhythmia in a Patient With Human Granulocytic Anaplasmosis: An Uncanny Presentation

**DOI:** 10.7759/cureus.13241

**Published:** 2021-02-09

**Authors:** Rana Raheel H Khan, Rabeea Zaki, Aiman Zaki, Javad Najjar Mojarrab, Ahmed Zahid

**Affiliations:** 1 Hospital Medicine, Marshfield Medical Center, Marshfield, USA; 2 Hospital Medicine, Marshfield Clinic Health System, Marshfield, USA; 3 Medical Oncology and Hematology, Almana General Hospital, Khobar, SAU; 4 Internal Medicine, Marshfield Clinic Health System, Marshfield, USA; 5 Internal Medicine, Marshfield Medical Center, Marshfield, USA

**Keywords:** human granulocytic anaplasmosis, atrial fibrillation

## Abstract

This is a case of a 69-year-old male, with well-controlled rheumatoid arthritis and benign prostatic hyperplasia, who presented with fever and generalized weakness. He was found to have atrial fibrillation on his second emergency department visit and later diagnosed with human granulocytic anaplasmosis (HGA). Atrial fibrillation subsided with the commencement of HGA-specific treatment. This is the first case of HGA and atrial fibrillation reported in the English literature. It highlights the importance of being vigilant for unusual presentations of tick-borne diseases in the endemic areas.

## Introduction

Human granulocytic anaplasmosis (HGA) is a rickettsial infection caused by the rickettsia Anaplasma phagocytophilum [1]. HGA is transmitted by the Ixodes scapularis ticks, and it is most prevalent in the Northeast and Upper Midwest United States. Patients usually present with nonspecific symptoms such as fever, chills, headache, myalgia, and malaise. Leukopenia, thrombocytopenia, and elevated liver enzymes are laboratory abnormalities commonly observed among patients infected with the HGA [2].

Most patients often are initially diagnosed as having a mild viral infection. However, as many as 3% of patients could develop life-threatening complications, and approximately 1% die from the infection. Thus, a high degree of clinical suspicion and a history of tick bite warrant early treatment with doxycycline in both adults and children. Early treatment resolves symptoms within 48 hours [2].

We present a case of a 69-year-old man who developed atrial fibrillation with a rapid ventricular response due to infection with HGA. Although arrhythmias are well-documented with Lyme disease, none have been reported with HGA. Atrial fibrillation is an unusual presentation of HGA. It has important implications in clinical practice regarding further testing and long-term anticoagulation.

## Case presentation

A 69-year-old male with a history of rheumatoid arthritis on weekly methotrexate and daily prednisone, diverticulosis, and benign prostatic hyperplasia (BPH) on tamsulosin presented to the emergency department with complaints of fever and generalized weakness of two days. According to the patient, he was in his usual state of health two days back when he started to have fever associated with chills, listlessness, and generalized fatigue. The highest recorded temperature was 102 °F. He denied having any cough, runny nose, sore throat, chest pain, difficulty breathing at rest, or exertion. He also denied having abdominal pain, nausea, vomiting or diarrhea, headaches, vision changes, photo or phonophobia, urinary burning, frequency, or urgency. He noticed that his urine had become dark and his urine output has decreased since the onset of fever. He reported a low appetite with a metallic taste attributed to dry mouth. He denied having any new skin lesions or rash, as well as any sick contacts. He had a tick bite three years ago but none noted recently. He admits to working in his backyard frequently and have his sauna in the woods.

He was brought to the emergency department for the evaluation of persistent high-grade fever. The temperature was 102.4 °F in the emergency department, blood pressure was 125/64 mmHg, heart rate was 73 beats per minute (bpm), and the respiratory rate was 18 breaths per minute (br/min) with oxygen saturation of 95% on room air. On physical examination, he was alert without any acute distress. There was no neck rigidity, the trachea was midline, and there was no cervical or inguinal lymphadenopathy. The oral mucosa was dry. No rash or petechiae were found. The first and second heart sounds were audible without any clicks or murmurs. The rhythm was regular. Lung, abdomen, neurological, and extremity exam was unremarkable.

**Figure 1:**
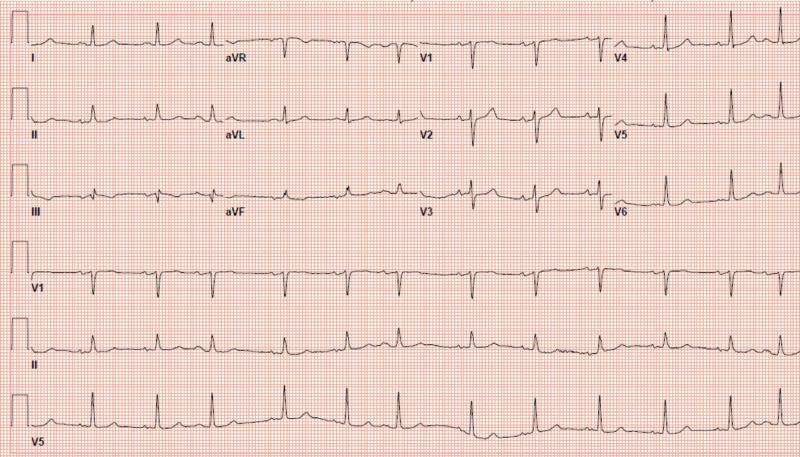
Twelve-lead electrocardiogram (EKG) at the time of the first emergency department presentation showing sinus rhythm with premature atrial complexes

Electrocardiogram (EKG) showed premature atrial contractions (Figure 1), and the chest X-ray was free of any acute pathology. Laboratory workup (Table 1), including complete blood count (CBC), basic metabolic panel, lactate, and liver function panel were unremarkable. He tested negative for severe acute respiratory syndrome coronavirus 2 (SARS-CoV-2). Inflammatory markers C-reactive protein (CRP) and procalcitonin were mildly elevated at 5.9 mg/dL (0.0-1.0 mg/dL) and 0.23 ng/dL (0.0-0.10 ng/dL), respectively. Urinalysis showed trace ketones and protein. Blood and urine cultures were both negative for any bacterial growth after five days. He received acetaminophen, his fever subsided, and he decided to go home from the emergency department, as he was feeling better.

**Table 1:** First and second visits lab results White blood cell (WBC); C-reactive protein (CRP); lactate dehydrogenase (LDH); thyroid-stimulating hormone (TSH); international normalized ratio (INR); activated partial thromboplastin time (APTT); aspartate aminotransferase (AST); alanine aminotransferase (ALT)

	ED Visit (First Visit)	Second Visit (Day 1)	Hospitalization Day 2	Hospitalization Day 3	Hospitalization Day 4	Hospitalization Day 5	Normal Range
WBC	5.5 x10^3/uL	3.1 x 10^3/uL	2.6 x 10^3/uL	3.3 x 10^3/uL		6.9 x 10^3/uL	4.1-10.9 x10^3/uL
Platelet	168 x 10^3/uL	56 x 10^3/uL	47 x 10^3/uL	48 x 10^3/uL		162 x 10^3/uL	150-450 x 10^3/uL
Hemoglobin	15.0 g/dL	15.8 g/dL	13.1 g/dL	12.5 g/dL		13.4 g/dL	12.9-17.3 g/dL
Hematocrit	43.8 %	45.3%	37.4 %	36%		38.4%	38-51.0 %
Albumin	4.0 g/dL	3	3.4 g/dL	3.1 g/dL	3.6 g/dL	3.2 g/dL	4.0-5.4 g/dL
AST	23 U/L	68 U/L	89 U/L	221 U/L	138 U/L	111 U/L	13-39 U/L
ALT	21 U/L	44 U/L	59 U/L	193 U/L	181 U/L	159 U/L	8-52 U/L
Alkaline phosphatase	75 U/L	149 U/L	129 U/L	141 U/L	135 U/L	109 U/L	43-115 U/L
Total bilirubin	1.3 mg/dL	2.2 mg/dL	1.4 mg/dL	1.1 mg/dL	0.7 mg/dL	0.7 mg/dL	0.1-1.0 mg/dL
CRP	5.9 mg/dL	17.9 mg/dL					0.0-1.0 mg/dL
Procalcitonin	0.23 ng/dL	1.6 ng/dL					0.0-0.10 ng/dL
Creatinine	0.9 mg/dL	0.9 mg/dL			0.8 mg/dL	0.8m mg/dL	0.6-1.2 mg/dL
Lactic acid	0.7 mmol/L						0.5-2.0 mmol/L
LDH		509 U/L	512 U/L		400 U/L	320 U/L	88-207 U/L
high sensitivity Troponin		20 ng/L				5 ng/L	0-57 ng/L
TSH		1.04 uIU/ml					0.55-4.78 uIU/ml
Acetaminophen level		11 ug/ml					0-10 ug/ml
INR		1.0	1.0	1.0	1.1	1.1	0.9-1.1
APTT		31.5 seconds					23.5-32.1 seconds

At home, he continued to spike fevers and took 1000 mg of acetaminophen every two to three hours, his fever did not subside, and he kept on feeling weak so decided to come back to the emergency room after two days. A review of systems was insignificant except for generalized weakness and dark-colored urine without burning, urgency, or frequency.

On physical examination, his temperature was 98.2 °F, heart rate was 105 bpm, blood pressure was 119/64 mmHg, respiratory rate was 17 br/min with oxygen saturation of 99% on room air. The skin was free of rash again. He was tachycardic with an irregular rhythm. The rest of the physical exam was unremarkable.

Laboratory workup (Table 1) showed thrombocytopenia with a platelet count of 56 x 10^3/uL (150-450 x 10^3/uL), elevated aspartate aminotransferase (AST) of 68 U/L (13-39 U/L), alkaline phosphatase of 149 U/L (43-115 U/L), and total bilirubin of 2.2 mg/dL (0.1-1.0 mg/dL). Lactate dehydrogenase LDH was elevated to 509 U/L (88-207 U/L). The hepatitis panel was negative. Urinalysis showed a specific gravity of 1.034, 1+ ketones, bilirubin presence, leukocyte esterase (1+), and nitrate positivity.

The respiratory viral panel along with SARS-CoV2 was negative again. The acetaminophen level was 11 ug/ml (0-10 ug/ml) and the urine toxicology screen was negative. The international normalized ratio (INR) was 1.0 with activated partial thromboplastin time (APTT) of 31.5 seconds (23.5-32.1 seconds). Electrolytes and renal function were within normal limits. CRP was 17.9 mg/dL (0.0-1.0 mg/dL) and procalcitonin trended up to 1.60 ng/dL (0.00-.10 ng/dL).

**Figure 2:**
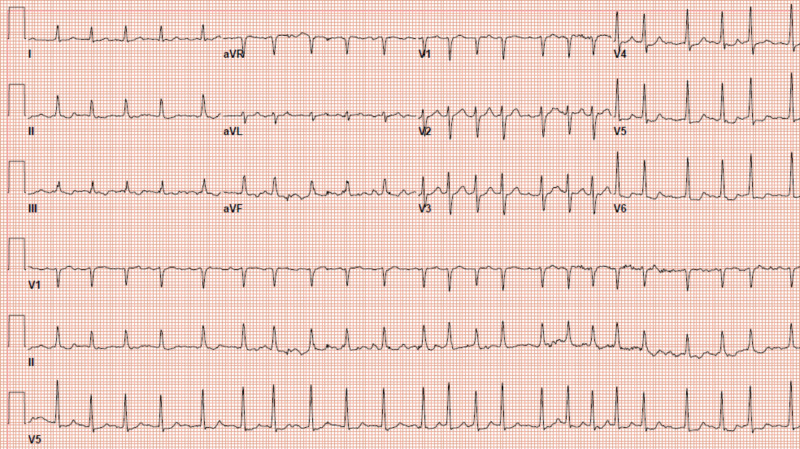
Twelve-lead electrocardiogram (EKG) at the time of hospitalization showing atrial fibrillation with a rapid ventricular response of 140 with nonspecific ST-segment changes

EKG showed atrial fibrillation (Figure 2) with a heart rate of 140 bpm. The chest X-ray did not show any acute pathology again, and a CT scan of the abdomen and pelvis was unremarkable.

The patient was started on a diltiazem drip for atrial fibrillation with a rapid ventricular response and received a ceftriaxone dose for possible urinary tract infection. He was also started on oral N-acetylcysteine (NAC) for acetaminophen toxicity after contacting the poison control center and admitted to the general medicine floor. The next morning, the peripheral blood smear showed Anaplasma phagocytophilum morulae and the ceftriaxone was switched to doxycycline. The polymerase chain reaction (PCR) was also positive for Anaplasma phagocytophilum; the rest of the tick-borne disease panel was negative, including Lyme serology, Ehrlichia, and Babesia. Blood cultures were negative and urine culture drawn on the second emergency department visit was positive for Enterococcus faecalis 10000-50000 org/ml.

INR and comprehensive metabolic panels were trended as per the recommendations of the poison control center. The liver enzymes peaked on the third day of hospitalization. Peak AST level was 221 U/L (13-39 U/L) and ALT was 193 U/L (8-52 U/L) with alkaline phosphatase of 141 U/L (43-115 U/L). The total bilirubin trended down to 1.1 mg/dL (0.1-1.0 mg/dL). INR remained stable at around 1.0 (0.9-1.1) throughout the hospital stay. On the fifth day of hospitalization, liver function tests (LFTs) started to trend down and at the time of discharge, the levels of AST, ALT, and alkaline phosphatase were 111 U/L (13-39 U/L), 159 U/L (8-52 U/L), and 109 U/L (43-115 U/L), respectively. The lowest white blood cell (WBC) count was 2.6 x 10^3/uL (4.1-10.9 x 10^3/uL), with a platelet count of 47 x 10^3/uL( 150-45 x 10^3/uL) on the second day of admission. White cell count improved to 6.9 x 10^3/uL (4.1-10.9 x 10^3/uL), and the platelet count was 162 x10 ^3/uL (150-450 x 10^3/uL) at the time of discharge. He completed 72 hours of oral NAC and was discharged home on oral doxycycline to complete a total of 14 days of treatment.

Regarding new-onset atrial fibrillation, his CHA2DS2-VASc (congestive heart failure, hypertension, age ≥ 75 years, diabetes mellitus, stroke or transient ischemic attack (TIA), vascular disease, age 65 to 74 years, sex category) score was one, and he was not started on anticoagulation. He converted to normal sinus rhythm on the second hospital day; Echocardiogram did not show any right or left ventricular wall motion abnormality; the calculated left ventricular ejection fraction was 65%. The right and left atria were mildly dilated, and there was no left atrial thrombus noted.

## Discussion

Human granulocytic anaplasmosis (HGA) is a rickettsial tick-borne infection caused by the Anaplasma phagocytophilum. HGA is transmitted by the Ixodes scapularis tick along the Northeastern and Upper Midwest regions and the Ixodes pacificus tick bite along the West coast of the United States [1-2]. It is an important cause of nonspecific febrile illness in Wisconsin during the summer season. The number of cases reported to the Centers for Disease Control and Prevention (CDC) increased steadily from 348 in 2000 to 5762 in 2017 in the United States; the cases reported in 2018 were substantially less [3]. The reported incidence of HGA is 6.3 cases per million individuals, and the case fatality is less than 1% [1-2,4].

Nonspecific febrile illness is the most common presentation of HGA. Other common clinical findings are malaise, headache, myalgia, and arthralgia. Less common presenting symptoms can be cough, nausea, and neck stiffness, and the least common presenting symptoms are confusion, diarrhea, vomiting, and rash. Severe complications, including septic shock, opportunistic infections, pancarditis, and polyneuropathies, have been reported in the literature [2,5-6]. Typical laboratory abnormalities include thrombocytopenia, leukopenia, and mild to moderate elevation of liver transaminases. Creatinine elevation and anemia have also been noted in many cases [1-2,5]. Peripheral smear may show morulae in the neutrophils in up to 20% to 100% of cases in the first week of illness and are diagnostic of HGA. The diagnosis can also be confirmed with PCR amplification of Anaplasma phagocytophilum deoxyribonucleic acid (DNA) from the blood. PCR has a better yield if done before the commencement of antibiotics. A four-fold increase in the antibody titers in paired samples (acute and convalescent) is the reference standard for diagnosis, but acute serology alone is insensitive (<50% sensitivity) [1-2,5,7].

The differential diagnosis of HGA is broad due to its nonspecific presentation and includes acute viral and bacterial infections as well as other tick-borne diseases. It can also be confused with thrombotic thrombocytopenic purpura, hematologic malignancy, and viral hepatitis [2,5,7].

Arrhythmias have been noted with Lyme disease [8], but no significant data is available related to HGA. In our literature review, we did not find any reported case of HGA associated with atrial fibrillation with rapid ventricular response or any dysrhythmias. However, HGA cases associated with myocarditis have been reported in the past [5-6]. We believe that this patient developed atrial fibrillation due to HGA infection without significant myocardium involvement, as the high sensitivity troponin remained within normal limits. One can argue that acetaminophen could be the potential cause, but its serum level was only slightly above the upper limit of normal. Acetaminophen has been reported to be associated with myocardial injury [9-10], causing dysrhythmias as a result. The postulated mechanisms of injury have been reported to be multifaceted, including metabolic disturbances, hypoxia, increased levels of free fatty acids, catecholamines, and hypotension. All have been secondary to hepatic failure as well as direct toxic effects like oxidative stress and structural changes in protein and gene derangements. All the reported cases failed to establish a causal relationship between cardiotoxicity and acetaminophen toxicity convincingly and stood out as mere associations [10]. However, the normal range of troponin in our patient will argue against acetaminophen, causing myocardial injury and, in turn, causing atrial fibrillation. Our patient also responded to the doxycycline treatment, and the atrial fibrillation subsided the next day of the admission. He remained in normal sinus rhythm during the rest of the hospitalization. It was a transient episode of atrial fibrillation. In the outpatient setting, he was placed on the cardiac event monitor for 30 days, and no further episodes of atrial fibrillation were noted.

## Conclusions

It is crucial to consider tick-borne illnesses in endemic areas in individuals presenting with viral-like nonspecific febrile illness. It is also important to note that they can have an unusual presentation and other factors contributing to laboratory data abnormalities like acetaminophen in our case. Atrial fibrillation can be present in patients with HGA transiently, and outpatient monitoring without anticoagulation can be considered in patients with low risk of stroke. In case the patient has a high CHADS2VASC score, regular anticoagulation guidelines should be followed with close subspecialty follow-up.
